# Substrate Induced Denitrification over or under Estimates Shifts in Soil N_2_/N_2_O Ratios

**DOI:** 10.1371/journal.pone.0108144

**Published:** 2014-09-22

**Authors:** Nicholas J. Morley, David J. Richardson, Elizabeth M. Baggs

**Affiliations:** 1 Institute of Biological and Environmental Sciences, University of Aberdeen, Aberdeen, United Kingdom; 2 School of Biological Sciences, University of East Anglia, Norwich, United Kingdom; Dowling College, United States of America

## Abstract

The increase in atmospheric nitrous oxide (N_2_O), a potent greenhouse and ozone depleting gas, is of serious global concern. Soils are large contributors to this increase through microbial processes that are enhanced in agricultural land due to nitrogenous fertilizer applications. Denitrification, a respiratory process using nitrogen oxides as electron acceptors in the absence of oxygen, is the main source of N_2_O. The end product of denitrification is benign dinitrogen (N_2_) and understanding what regulates the shift in ratio of N_2_O and N_2_ emission is crucial for mitigation strategies. The role of organic carbon in controlling N_2_O reduction is poorly understood, and mostly based on application of glucose. Here we investigated how a range of carbon compounds (succinate, butyrate, malic acid, acetate, glucose, sucrose and cysteine) affect denitrifier N_2_/N_2_O production stoichiometry under laboratory conditions. The results show that a soil's capability in efficiently reducing N_2_O to N_2_ is C substrate dependent and most compounds tested were different in regards to this efficiency compared to glucose. We challenge the concept of using glucose as a model soil C compound in furthering our understanding of denitrification and specifically the efficiency in the N_2_O reductase enzyme. Organic acids, commonly exuded by roots, increased N_2_/N_2_O ratios compared to glucose, and therefore mitigated net N_2_O release and we suggest provides better insights into soil regulatory aspects of N_2_O reduction. The widespread use of glucose in soil laboratory studies could lead to misleading knowledge on the functioning of denitrification in soils with regards to N_2_O reduction.

## Introduction

Globally soils are the largest anthropogenic source of the greenhouse gas nitrous oxide (N_2_O) [1], which is increasing in atmospheric concentrations by ∼0.25% per year, has a radiative forcing ∼300 times that of carbon dioxide and plays a major role in stratospheric ozone depletion [2]. The increases in anthropogenic emissions present serious concerns for future global climate and environmental changes. Denitrification is a microbial process which sequentially reduces nitrates (NO_3^−^_) to dinitrogen (N_2_) via nitrite (NO_2^−^_), nitric oxide (NO) and N_2_O utilizing organic carbon (C) as the electron donor; a form of respiration in the absence of oxygen [3]. Denitrification is the predominant biological process responsible for global increases in atmospheric N_2_O [4] mainly due to the large inputs of nitrogenous-based fertilizers to arable land to meet or increase food productivity. Understanding the regulation of denitrification and the role of organic C in the production of N_2_O and its reduction to N_2_ is of global importance and offers the opportunity for managing soils to lower net emission of N_2_O.

Product stoichiometry of N_2_ and N_2_O is an important parameter for denitrification as it controls whether N_2_O or the benign N_2_ is released to the atmosphere. Increasing the N_2_/N_2_O ratio essentially mitigates the harmful effects of N_2_O [5]. The form and quantity of C utilised by soil denitrifiers can control the rate or efficiency of the N_2_O reductase enzyme altering product ratios of the emitted gases [6]–[8]. This is of particular relevance to soils as the ratio of N_2_/N_2_O emissions is directly influenced by environmental conditions such as the type of C substrates being exuded by plant roots within the rhizosphere. The regulation of denitrification by C is poorly understood compared to other soil parameters such as pH, N availability, redox status and water content [9]. A common practice in laboratory-based soil studies investigating process rates (denitrification potential, denitrification enzyme activity and N_2_/N_2_O emissions) is the application of an exogenous organic C substrate to stimulate heterotrophic denitrification, often referred to as “substrate induced”. This practice has been applied for mechanistic studies in order to understand how certain environmental conditions (e.g. water, temperature, pH and N availability) regulate denitrification rates, N losses and the stoichiometric ratio of N_2_/N_2_O production. The C substrate of choice appears to be glucose [10]–[21], and current understanding of the regulation of N_2_O reduction by C is principally based on glucose amendment to soil. Yet, the complexities of low molecular C compounds in soils, especially within the rhizosphere, that are available to the microbial population go beyond the model compound glucose. In order to advance understanding with a more realistic perspective other C substrates need to be considered to elucidate the unknown responses of these compounds on denitrifier product stoichiometry as would occur in the rhizosphere. In addition, glucose is the substrate used in the denitrification enzyme activity assay or to measure the denitrification potential for a given soil and set of conditions [22]. These techniques measure the potential rate of microbial denitrification at a given time by removing all limiting factors such as oxygen, NO_3^−^_ and glucose-C. However, use of glucose alone would not provide a complete oversight or predictor of the regulatory factors controlling ecosystem functioning (N_2_/N_2_O ratios) *in situ*. Studies examining denitrification product ratios with the addition of an exogenous C substrate require cautious interpretation, as the stoichiometry could differ depending on the nature of the compound applied to a soil [6], [7]. This has clear implications for our understanding of the effects of N_2_O on our environment.

In this study we assessed how addition of individual organic C substrates (glucose, sucrose, acetate, malic acid, butyrate, succinate and cysteine) and no external C, here termed soil organic matter-carbon (SOM-C), affected soil N_2_ and N_2_O production kinetics, using ^15^N-labelled NO_3^−^_ tracer techniques for quantification of denitrification stoichiometries. Specifically, we were interested in the extent to which different substrates deviated in denitrification stoichiometries from the model glucose. We hypothesised that different C substrates would result in differences in the efficiency of N_2_O reduction to N_2_, with higher N_2_/N_2_O ratios with the addition of organic acids (acetate, butyrate, succinate and mailc acid) compared to sugars and an amino acid, due to the ease that organic acids can be metabolically converted for entry into the tricarboxyl acid cycle [23].

## Materials and Methods

### Soil

Soil (0–15 cm depth) was sampled from a pasture site near Insch, Aberdeenshire (N.E. Scotland, 57°33′ N; 2°63′ W), classified as a Dystric Cambisol. The sampling site did not involve endangered or protected species and no written permissions or permits were required to access or sample the site. Insch soil has a sandy loam texture (sand 57.7%, silt 30.8%, clay 11.5%), pH (H_2_O) 6.1 and a total organic C content of 51.3 mg C g^−1^ soil. Once brought to the laboratory the soil was sieved (≤4 mm) with any visible plant material removed.

### Experimental design and treatments

Soil (260 g at 24% gravimetric water content) was packed into PVC cores (diameter 5 cm and height 30 cm) to a bulk density of 1 g cm^−3^ with a 10 cm soil profile. Cores were fitted with a headspace gas sampling port and could be sealed with PVC caps creating a gas tight headspace. The soil cores were wetted with de-ionised H_2_O (dH_2_O) to a target water-filled pore space (WFPS) of 70% and the mass of individual cores recorded. Cores were left to pre-incubate in the laboratory in the dark (temperature range 19–22°C) for 6 days. After 6 days dH_2_O was added to each core to re-adjust the cores to their original mass at 70% WFPS, to account for any water loss by evaporation during pre-incubation. To apply organic C substrates to cores a Micro-Rhizon (Rhizosphere Research Products) tube (2.5 mm diameter, 10 cm porous section) was inserted into the centre of each core vertically along the entire soil profile. The top end of the Micro- Rhizon tube was connected to PVC manifold tubing and loaded using cassettes onto a 32 channel peristaltic pump (205S, Watson and Marlow). This design allowed for 32 soil cores to be simultaneously connected to the peristaltic pump for solution to be applied into the soil along the porous section of the Micro-Rhizon tube.

24 hours after insertion of the Micro-Rhizon tube into the soil ^15^N-NO_3^−^_ was applied evenly to the surface of the soil in a 4 ml dose from a sterile solution of K^15^NO_3^−^_ (422 mM at 30.3 atom% excess ^15^N) resulting in a final soil N application rate of 23.6 mg N to each core (equivalent to 119.2 µg N g^−1^ soil; or 12 g N m^−2^). After ^15^N application a further 3 ml H_2_O was added to the soil surface, raising the soil WFPS to 80%. Subsequently the peristaltic pump was switched on (rotor speed 0.5 r.p.m) and the manifold tubing inserted into sterile reservoirs of either organic C stocks (glucose, sucrose, acetate, malic acid, succinate, butyrate or cysteine) or water. The pump was initially left on for 4 hours to allow the liquid flow to reach the Micro-Rhizon tube, but subsequently the pump was controlled by an electronic timer set to a 2 hour ON/OFF cycle for 14 days. This ON/OFF pumping cycle resulted in a total flow rate of 2.2 ml day^−1^ (standard error ±0.05) through the tube into the soil. The flow rate of the peristaltic pump and C reservoir concentrations resulted in a soil C application rate of 15 mg C day^−1^ core^−1^ (standard error ±0.33) equivalent to 76.6 µg C g^−1^ soil for all compounds. Each treatment was replicated 4 times for soil gas measurements and addditional cores for each treatment were used for destructive soil sampling (n = 3, per treatment per sample point) at 3 and 7 days after ^15^N application. An additional 3 cores were used for day 0 soil samples (immediately after ^15^N application but not connected to pump) and gas measurement cores were destructively sampled for soil immediately post gas sampling on day 14.

### Soil headspace gas measurements

In order to determine soil gas production rates soil cores were sealed with gas tight plastic caps. A preliminary test showed that the increase in CO_2_ and N_2_O headspace concentrations were linear over the 1 h closure period. Ambient concentrations were determined from laboratory air samples at t  = 0, with gas sampling from core headspaces performed between 12:00 and 14:00 daily for initially 7 days then either every 2 or 3 days for a further 7 days. After 1 hour headspace closure, 15 ml gas samples were removed with a syringe fitted with a SGE syringe valve and transferred into pre-evacuated gas vials (Labco Ltd. UK). Immediately after, a second larger gas sample (125 ml) was withdrawn from the headspace using a 0.5 L Jumbo gas tight syringe (SGE) fitted with a valve. The larger gas samples were transferred into pre-helium flushed evacuated amber storage bottles (Supelco) fitted with silicone/PTFE septa (Supelco). Gas samples were stored in the laboratory in the dark for no longer than 2 weeks prior to analysis.

Gas samples were analysed for ^14+15^N-N_2_O concentrations on an Agilent 6890 gas chromatograph fitted with an Electron Capture Detector (detector temperature 350°C). Chromatographic separation was achieved using a Hayesep Q column (60°C) with nitrogen as a carrier gas (25 ml min^−1^). Chromatographic peaks were integrated and converted into concentrations (µl L) following calibration with known gas standards. The ^15^N isotopic enrichments of N_2_O and N_2_ were determined on a 20–20 continuous flow isotope ratio mass spectrometer (SerCon Ltd.) following cryofocusing of the gas sample in an ANCA TGII trace gas preparation module. Atmospheric air was used as an instrumental ^15^N reference. Atom% ^15^N values of N_2_O were calculated using drift corrected signal ratios *m/z* 45/44 and 46/44 following ^17^O and ^18^O oxygen corrections according to Bergsma *et al.* [24], due to the occurrence of natural abundance of O isotopes in the N_2_O molecule.

### Soil measurements

Additional replicate soil cores (attached to the peristaltic pump) were destructively sampled for soil on days 0 (immediately after N application), 3, 7 and 14 (using gas measurement cores). Soil from an entire core was placed in a polythene bag and thoroughly homogenised prior to subsamples being taken for analysis. Soil moisture content was determined gravimetrically by drying in an oven at 105°C for 24 hours. Soil NO_3^−^_ and NO_2^−^_ concentrations were determined colorimetrically on a Burkard SFA2 autoanalyser after extraction with 1 M KCl.

### Calculations and statistical analyses

N_2_O and N_2_ production rates are expressed herein as mg N (N_2_O or N_2_) core^−1^ as cumulative production over time. Soil denitrification N_2_/N_2_O ratios were calculated from the cumulative ^15^N-N_2_O and -N_2_ production. To measure the denitrifier N_2_O and N_2_ contribution to measured gases in headspace ^15^N-N_2_O and ^15^N-N_2_ atom% excess values from samples were calculated by subtracting corresponding ^15^N atom% values of N_2_O and N_2_ measured from isotopically un-enriched (natural abundance) reference soil cores. The proportion of N gas in the headspace that was derived from the applied ^15^N-NO_3^−^_ was calculated according to the method described by Bergsma *et al.* [24] and Equation (1), by taking into account the isotopic enrichment of the NO_3^−^_ substrate: 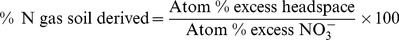
(1)


To test whether there were differences between C treatments in N_2_/N_2_O ratios we performed linear regressions of N_2_/N_2_O ratios over time, and to show that the slopes were significantly different we performed ANCOVA (Matlab Mathworks 2013) followed by a Tukey-Kramer [25] multiple comparisons of slopes at a significance level of 0.05.

## Results and Discussion

**Figure 1:**
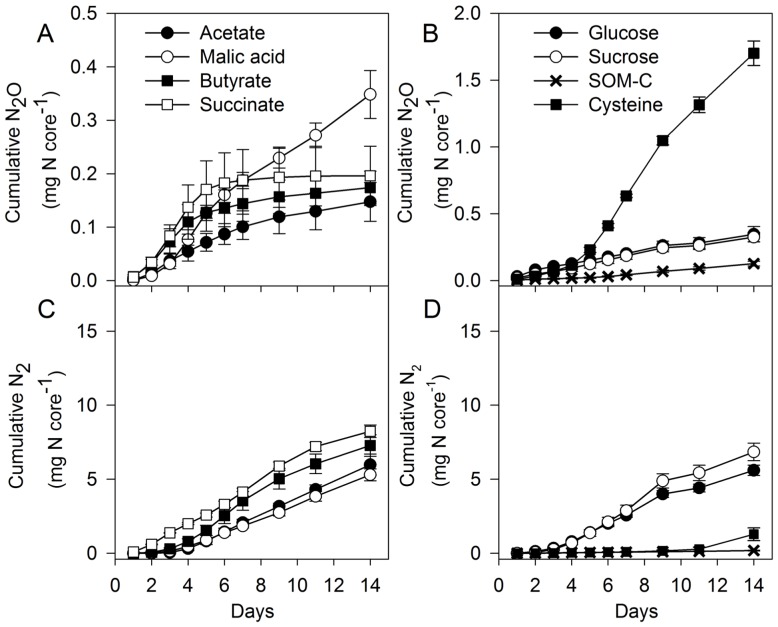
Cumulative ^15^N-N_2_O and ^15^N-N_2_ gas productions derived from **Equation (1**) from soil cores supplied with different forms of organic substrates or none (SOM-C). (A) N_2_O-N and (C) N_2_-N gas productions for acetate, malic acid, butyrate and succinate treatments. (B) N_2_O-N and (D) N_2_-N gas productions for glucose, sucrose SOM-C and cysteine treatments. Values are means ±1 SEM (n = 4). The SOM-C treatment is the pooled results from 4 separate consecutive SOM-C experiments (n = 16).

We measured soil denitrification N_2_O and N_2_ gas production in soil cores that were semi-continuously supplied with organic substrates in the laboratory using a ^15^N- NO_3^−^_ tracing technique. Fig. 1 illustrates the cumulative denitrifier gas production kinetics over the 14 day experiment. The trends in gas production rates are common to other soil denitrification studies, with N_2_O being dominant as a product in the initial stages of the experiment and N_2_ production showing a classical delayed induction [26]–[29]. However, there were clear differences in these trends between the different C treatments. Succinate induced the greatest instant soil N_2_ production, as shown by others [30], with a direct link between the electron transport chain and the denitrification respiratory system and hence providing an instantaneous source of electrons to the denitrifying microbial pool. However, with all the other C substrates N_2_ production rates were slow initially and rates only increased after 3 to 4 days after addition of ^15^N. The exception to this were soils which were amended with cysteine or water only (SOM-C) that produced very small quantities of N_2_ throughout, and N_2_O was the dominant denitrifier product in these treatments. This suggests that the soil organic matter carbon was limiting for denitrification, and also lowered the efficiency in N_2_O reduction to N_2_, which from previous studies has been shown to be correlated to soil organic C content [31]. The amino acid cysteine treatment was different in that upon mineralization it releases hydrogen sulphide, which is known to inhibit denitrification [32] and especially the N_2_O reductase enzyme [33], here resulting in 4 fold more N_2_O production than all other treatments. In the three organic acid treatments (succinate, butyrate and acetate) N_2_O production almost ceased 7 or 8 days after the start of experiment, whereas all the other C amended soils continued to produce N_2_O throughout the experiment. This suggests a highly efficient denitrification pathway with some organic acids in which all reaction steps are functioning at equal rates, that has clear environmental benefits. An efficient denitrification system would enhance reduction of N_2_O to N_2_, and therefore mitigating N_2_O release to the atmosphere.

**Figure 2:**
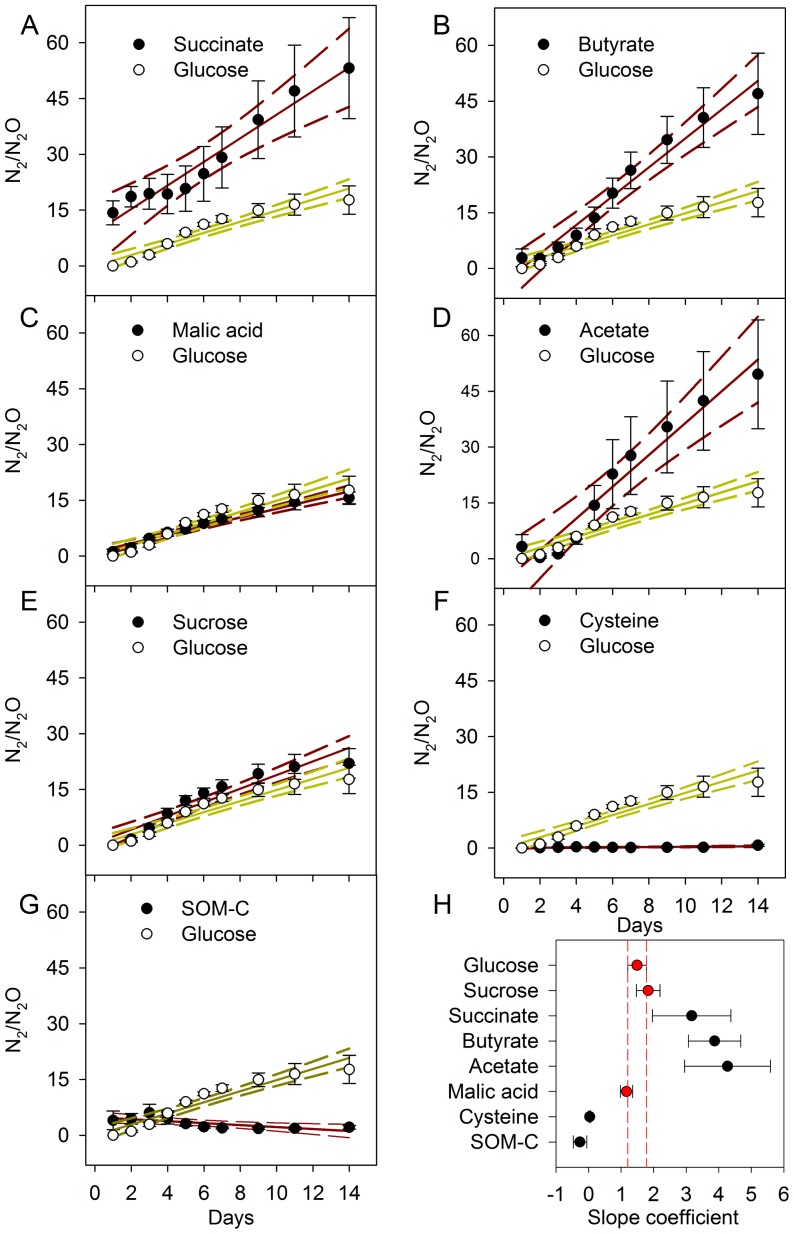
The N_2_/N_2_O ratios from the cumulative productions of ^15^N-N_2_ and ^15^N-N_2_O over time and slope coefficients form linear regressions. (A–G) N_2_/N_2_O ratios for each treatment plotted alongside glucose for pair wise comparisons. Solid lines are linear regression, with 95% confidence bands (dashed lines). Values are means ± SEM (n = 4) and SOM-C n = 16. (H) Shows the slope coefficients (errors bars are ±95% confidence intervals) from regression analysis. The dashed red lines show the confidence intervals of the glucose treatment and the red symbols represent slopes which are not significantly different from glucose (ANCOVA and Tukey Kramer multiple comparison).

To make comparisons in the efficiency of denitrification in terms of N_2_O reduction to N_2_, we examined N_2_/N_2_O ratios over time (Fig. 2), and applied linear regression models to the data. We were specifically interested in ratio differences between substrates when compared to glucose in a pair wise manner, as glucose is the most commonly applied compound for determining substrate induced soil processes. To test for differences between treatments we used ANCOVA (C treatment as main effect and time as a covariate) to compare the slope coefficients from linear regression models. The result from the ANCOVA revealed that N_2_/N_2_O slope coefficients were not homogenous between the different treatments (assessed by the interaction term C-treatment*Time; F = 41.5, df = 7 *P*<0.01), implying that the efficiencies of denitrification are not constant between the different organic substrate amendments. Following this we pair wise compared regression slopes to the glucose treatment using a Tukey-Kramer multi comparison procedure. The results revealed that acetate, succinate, butyrate and the SOM-C N_2_/N_2_O slope coefficients were significantly different from glucose at *P*<0.01, and the cysteine treatment was different from glucose at *P*<0.05 (Fig. 2). The regression slopes from sucrose and malic acid were not significantly different from that of glucose. This is visually presented in Fig. 2H, which shows that slope coefficients of N_2_/N_2_O ratios over time were mostly dissimilar to glucose amended soils, highlighting that the efficiency in the N_2_O reductase can be C compound type dependent. The greatest slope differences measured from glucose were 2.78, 2.38 and 1.68 with acetate, butyrate and succinate, respectively. These results suggest that soil microcosm studies, in which glucose has been applied to measure denitrifier stoichiometry would have measured various responses with different substrate amendments, shedding little actual light on the regulation of the N_2_O reductase. Glucose may not be the most appropriate substrate for understanding the regulation of N_2_O reductase and the regulation of N_2_O reduction to N_2_ is not possible to predict from glucose or sugar group of C compounds acting as the electron donor. Here organic acids tended to increase N_2_O reductase efficiency compared to carbohydrates and so we propose that organic acid substrate induced denitrification could provide more appropriate insights into the regulation of N_2_O reduction to N_2_ in soil. The results clearly demonstrate that a soil's denitrification response and efficiency in the reduction of N_2_O to N_2_ is C substrate dependent and conclusions drawn from such experiments in determining the regulation of N_2_O reduction would depend on the C substrate used as an inducer of denitrification.

Although only one soil was assessed here, we can expect that responses would vary between different soil types, due to differing quantities and composition of native SOM and different native microbial communities. Whilst beyond the scope of this study, substrate utilization preferences between different groups of microorganisms, competition and substrate diffusivity in soil could all be expected to affect denitrification rates (and N_2_/N_2_O ratios). More experimental work would be required to establish the importance of such indirect effects of substrates on denitrification and product stoichiometry. We found no consistent influence of soil pH or nitrate availability in regulating differences in N_2_/N_2_O ratios, suggesting a more direct substrate response supporting the hypothesis that C substrates can directly affect N_2_/N_2_O ratios by changing the efficiency in N_2_O reduction to N_2_. In substrate amended cores soil pH on day 14 was only significantly different between some treatments (Table S1) and so differences in soil pH due to substrate amendments alone could not explain measured differences in N_2_/N_2_O ratios. Soil NO_3^−^_ concentrations decreased throughout the experiment, but final concentrations on day 14 were only significantly (*P*<0.05) different in the SOM-C and cysteine treatments, and soil NO_2^−^_-N concentrations only increased in the succinate and butyrate treatments (∼4 µg N g^−1^ soil) (Fig. S1).

Reduction of N_2_O to N_2_ was most efficient with organic acids succinate, acetate and butyrate as shown by the largest slope coefficients in these C-amended soils (Fig. 2). From slope differences N_2_/N_2_O ratios compared to glucose were enhanced by 112%, 160% and 186% in the presence of succinate, butyrate and acetate, respectively, but lowered in comparison to glucose by 98% and 118% in the presence of cysteine or the native SOM-C for this soil. This different regulation of N_2_O reduction opens the opportunity to explore using root chemical traits or plant breeding to lower N_2_O emission to the atmosphere by increasing the N_2_/N_2_O ratio through a specific or altered rhizodeposition. Organic acids - major constituents of labile root carbon exudation [34], [35] - may be key to achieving this.

## Supporting Information

Figure S1 **NO_3^−^_-N and NO_2^−^_-N concentrations in soil cores supplied with different forms of organic substrates or none (SOM-C).** (A) NO_3^−^_-N and (C) NO_2^−^_-N concentrations in acetate, malic acid, butyrate and succinate treatments. (B) NO_3^−^_-N and (D) NO_2^−^_-N concentrations in glucose, sucrose, SOM-C and cysteine treatments. Values are means ±1 SEM (n = 3). The SOM-C treatment is the pooled results from 4 separate consecutive SOM-C experiments (n = 12).
(DOCX)

Table S1 **Soil pH in cores supplied with different forms of organic substrates or none (SOM-C).** Values are means (n = 3) calculated from H^+^ concentration and the minimum-maximum values in parentheses. Different letters indicate significant (*P*<*0.05*) differences between treatments on specific days.
(DOCX)
