# Supplementary figures and images for: Substrate Induced Denitrification over or under Estimates Shifts in Soil N2/N2O Ratios
Source: PLoS One. 2014 Sep 22;9(9):e108144. doi: 10.1371/journal.pone.0108144 (PMC4171533; doi:10.1371/journal.pone.0108144)

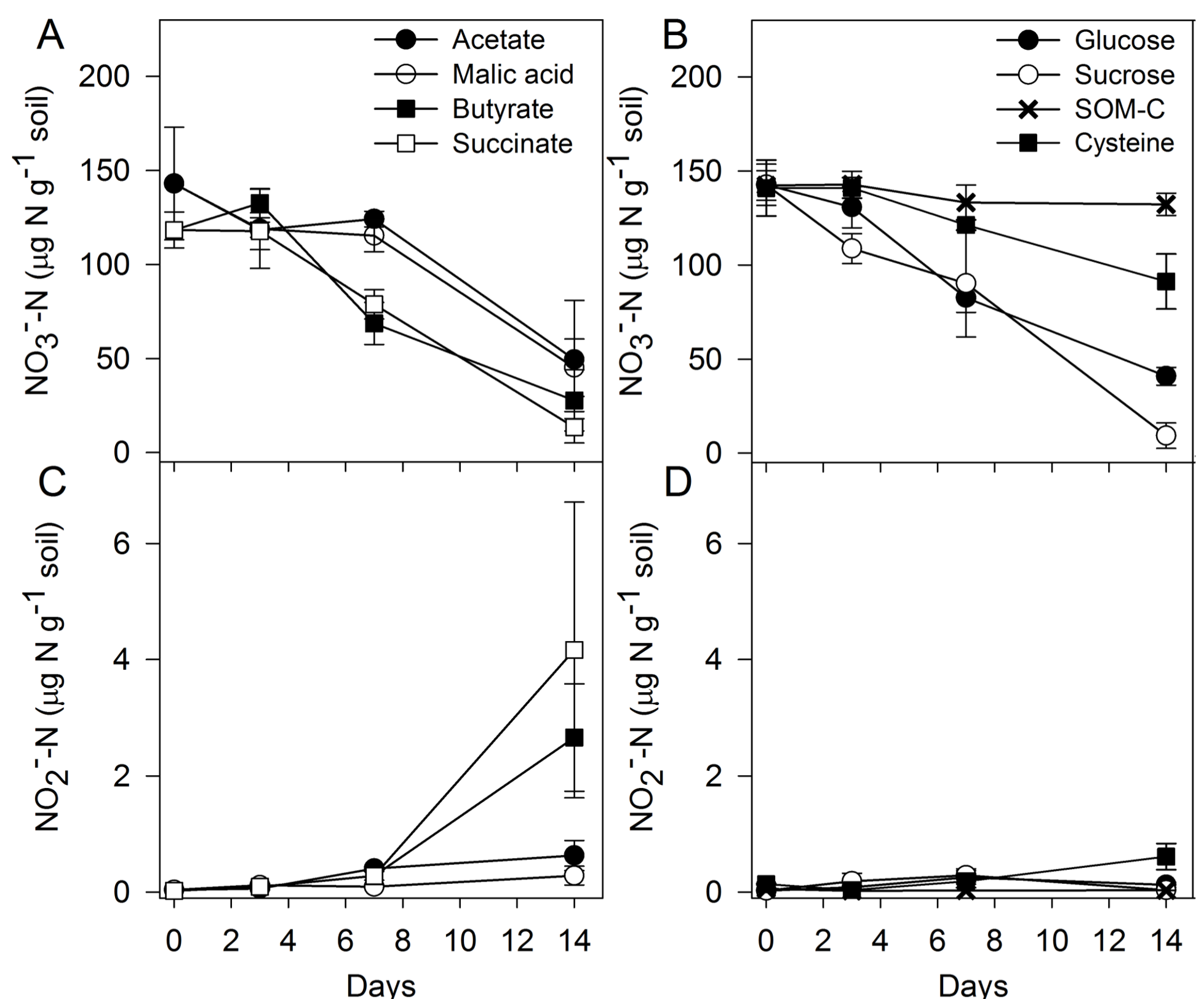

Supplement: Figure S1 — NO3−-N and NO2−-N concentrations in soil cores supplied with different forms of organic substrates or none (SOM-C). (A) NO3−-N and (C) NO2−-N concentrations in acetate, malic acid, butyrate and succinate treatments. (B) NO3−-N and (D) NO2−-N concentrations in glucose, sucrose, SOM-C and cysteine treatments. Values are means ±1 SEM (n = 3). The SOM-C treatment is the pooled results from 4 separate consecutive SOM-C experiments (n = 12). (DOCX) [file pone.0108144.s001.docx]
