# Supplementary material for: Substrate Induced Denitrification over or under Estimates Shifts in Soil N2/N2O Ratios
Source: PLoS One. 2014 Sep 22;9(9):e108144. doi: 10.1371/journal.pone.0108144 (PMC4171533; doi:10.1371/journal.pone.0108144)
Supplement: Table S1 — Soil pH in cores supplied with different forms of organic substrates or none (SOM-C). Values are means (n = 3) calculated from H+ concentration and the minimum-maximum values in parentheses. Different letters indicate significant (P<0.05) differences between treatments on specific days. (DOCX) [file pone.0108144.s002.docx]

**Table S1. Soil pH in cores supplied with different forms of organic substrates or none (SOM-C).** Values are means (n = 3) calculated from H^+^ concentration and the minimum-maximum values in parentheses. Different letters indicate significant (*P<0.05)* differences between treatments on specific days.

| Day | Glucose | Sucrose | Acetate | Malic acid | Butyrate | Succinate | Cysteine | SOM-C |
| --- | --- | --- | --- | --- | --- | --- | --- | --- |
| 0 | 6.22^a^  (6.2-6.3) | 6.22^a^  (6.2-6.3) | 6.07^a^  (6.0-6.2) | 6.07^a^  (6--6.2) | 6.37^a^  (6.4-6.4) | 6.37^a^  (6.4-6.4) | 6.34^a^  (6.3-6.4) | 6.23^a^  (6.0-6.4) |
| 3 | 6.32^ab^  (6.3-6.4) | 6.27^ab^  (6.1-6.4) | 6.26^ab^  (6.1-6.6) | 6.09^a^  (6.0-6.2) | 6.41^ab^  (6.4-6.5) | 6.56^b^  (6.4-6.8) | 6.56^b^  (6.5-6.7) | 6.14^a^  (5.8-6.3) |
| 7 | 6.34^a^  (6.3-6.5) | 6.27^a^  (6.2-6.7) | 6.44^a^  (6.3-6.8) | 5.87^b^  (5.9-5.9) | 6.33^a^  (6.2-6.6) | 6.54^a^  (6.5-6.7) | 6.50^a^  (6.4-6.7) | 6.2^a^  (6.0-6.4) |
| 14 | 6.47^ab^  (6.5-6.7) | 6.84^a^  6.7-6.9 | 6.41^a^  (6.3-6.6) | 6.24^bc^  (6.2-6.3) | 6.41^ab^  (6.3-6.6) | 6.52^ab^  (6.4-7.1) | 6.28^bc^  (6.1-6.5) | 6.13^c^  (6.0-6.3) |
